# The British Journals, &c.: Surgery

**Published:** 1842-10

**Authors:** 


### SURGERY.

Malignant Diseases of the Head and Face. The question naturally arises what are the symptoms of osteo-sarcoma of a malignant nature, and how are we to ascertain the precise time when the change from benign to malignant takes place. The following inferences are deducible from what is known of osteo-sarcoma of the lower jaw-bone. 1st. The disease almost always commences in the cancellated structure of the bone, and has generally a cystic origin, which it maintains from first to last. 2d. It is always (as far as recorded cases permit us to judge) mild under the ages of twenty-eight or thirty years; and although it does not necessarily become malignant after these periods, yet it generally does, involving the soft parts, and assuming the characters of carcinoma. 3d. Osteo-sarcoma of the lower jaw is almost always curable by free excision, before the soft parts become involved in the disease, which they never do in the benign form, nor for several months after the disease has assumed the carcinomatous form. 4th. Cancer of the face, especially the bones, admits of cure more frequently than when seated in any other part of the human frame; cancer scroti, chimney sweeper's cancer, perhaps excepted. 5. Ligature of the carotid is unncessary previous to or during the disarticulation of the jawbone.—Dr. Byron; *Dublin Journal of Med. Science, July*, 1842.

---

Displacement of the Stomach and Colon in consequence of a Wound. A serjeant of the 88th regiment, when skirmishing on the day previous to the battle of Fuentes d'Onor, was fired at as he was attempting to ascend a steep hill, the man who fired at him being at the top. The ball entered close to the nipple of the left breast, and passed out at the back, between the eighth and ninth ribs. The man recovered, rejoined the service, and died of gangrene of the lower limb in 1833.

On examining his body, the anterior surface of the stomach was found firmly attached to the lower lobe of the left lung. It would appear that the whole of the stomach and greater part of the transverse colon, with the omentum, had passed into the left cavity of the thorax. The heart was displaced so as to lie nearly parallel to the spine, its apex almost on a line with the coronary artery of the liver. The wound in the diaphragm, through which the hernia of the stomach and other parts had taken place into the thorax, appeared to have extended originally about three inches.—G. Williamson, Esq., *Medical Gazette, July* 15, 1842.

CASE OF OSSEOUS ANEURISM. The patient, aged nineteen, had injured the right knee, near the inner condyle, by slipping over a stone bank, five months before Mr. Hargreaves saw him. Five weeks after the accident, the patient observed a slight swelling on the inner side of the knee-joint, occasionally communicating to the touch, "a degree of beating." The tumour had now attained an enormous magnitude; measuring round the knee-joint, twenty-four inches. It was accompanied with diffused hardness, and extended as high as the inferior third of the thigh, filling up the whole popliteal space. A very indistinct pulsation could *sometimes* be heard at the internal condyle, but neither pulsation nor tremor was communicated to the touch.

The femoral artery was tied on the 11th of April last. The case appeared to go on well till the 28th, when there was epistaxis of an apparently obstinate character; and an ichorous discharge commenced from the ulcerated integuments, [around the wound we presume,] and a sensation of fluctuation was felt. The limb also gradually enlarged. A consultation was held as to the propriety of removing the limb; but this step was deemed inadmissible, as petechiæ appeared on the affected member, and spread rapidly over the body, while hemorrhage took place incessantly from the mouth and fauces. He died on the 1st of May.

On making an incision, and exposing the popliteal space, a sac was exposed, extending as high as the middle third of the thigh; this sac was full of sanguineous fluid, and contained a semi-cartilaginous substance; interspersed with a little ossific matter. It was difficult to say, whether the sac itself was composed of the periosteum, or had a distinct origin, but the tendons, muscles, nerves, and blood-vessels, did not present any abnormal appearance. The popliteal artery, vein and nerve, were situated very superficially, and upon the sac, which had not communicated with the artery; this vessel being perfectly sound.—Mr. HARGREAVES, Lancashire; *Medical Gazette*, No. 757, *June* 3, 1842.

---

MR. STAFFORD'S TREATMENT OF STRICTURE OF THE URETHRA. The single lancetted stilette, or urethral perforator, is passed down to the stricture, the exact distance of which from the extremity of the urethra is first ascertained. When the point of the instrument is arrived at and rests upon the contraction, (which is known by means of its graduation,) and is in an exact line with the natural course of the canal, the instrument is held and maintained in that position by the left hand, the forefinger of which being passed through the ring on the under part of its handle, the thumb of the right hand is passed through the ring on the handle of the stilette. The stilette is then pressed gently and gradually forward, *in the direction of the canal,* when, on reaching and resting on the stricture, the lancet is protruded at its point, and is thus made to incise the stricture, immediately after which the bougie or catheter may be passed with facility.—WILLIAM COULSON, Esq., Surgeon to the Magdalen Hospital; *Med. Gazette,* No. 763. *July* 15, 1842.

---

MORPHIA IN STRANGULATED HERNIA. Mr. Lyell records a successful case. Within three hours, the patient had three grains of opium, and four and a half of hydrochlorate of morphia, and the rule which Mr. Lyell would recommend is, *to employ the morphia in half grain hourly, or half-hourly doses, till the patient is fairly narcotised.*—JOHN LYELL, Esq., Fife; *Lond. and Edin. Journ. of Med. Science,* No. vii. *July,* 1842.

---

MOXA IN RHEUMATISM. The writer reports excellent effects from the use of the moxa among the labouring poor of Ireland, who appear to be very subject to local rheumatism, in consequence of exposure and insufficient clothing. Mr. Leney soaks a piece of lint in a strong solution of nitrate of potass, then dries it, and cuts off pieces the size of the thumb-nail, which he fastens with thin adhesive plaster over the seat of pain, sets fire to the opposite extremity, and then applies the blowpipe. The pain during the operation is very severe, but the Irish prefer it much to the application of blisters!—JOHN LENEY, Esq. Bray; *Med. Gazette,* No. 763, *July* 15, 1842.

TINCTURE OF CATECHU IN SORE NIPPLE. Mr. Farr highly recommends the tincture of catechu in sore nipple. It has the effect of "thickening and toughening the nipple and surrounding integument." Messrs. Hopgood of Bampton, Devon, and Pye, H. Chavasse of Birmingham, confirm Mr. Farr's account of the uncommon efficacy of the remedy.—WILLIAM FARR, Esq. of the General Register Office; *Lancet*, No. xv. *July* 9, 1842. [The nipple is first to be washed and dried, and then the tincture applied with a hair pencil. In No. xvii. of the same journal (July 23) Mr. Wansbrough of Chelsea recommends "the leaden shield," which is "moulded by the plumber from thin sheet lead, over a wooden nipple, and forms a receptacle for the natural one, which, from the oozing of the breast, is constantly immersed in a solution of the lactate of lead, and speedily effects a cure."]

